# To the Medical Profession and Scientific Observers

**Published:** 1857-01

**Authors:** 


### MEDICAL INTELLIGENCE.

*To the Medical Profession and Scientific Observers.*

The undersigned having been appointed, at the late meeting of the American Medical Association, Chairman of a Committee on the Etiology and Pathology of Epidemic Cholera, respectfully solicits Etiological, Pathological, and Historical data pertaining to the disease, from the medical profession, and Meteorological data from any person who may have them.

As a primary examination in investigating the cause of Asiatic Cholera and its mode of travel, he wishes to be able to compare Meteorological data obtained when the disease was raging in any given locality with those which were obtained where the disease did not prevail.

To make this comparison most complete, a full set of Barometrical, Thermometrical, Hygrometrical, and Ozonometrical tables, with prevailing winds and electrical phenomena, as far as obtainable, for a series of years, embracing those of health and disease, and extending over a large territory, are needed; but where full tables are not obtainable, partial ones, or reports, will be thankfully received if the observations are accurate.

As Hygrometrical observations are extremely rare in the United States, the undersigned hopes that each person who has any which may be of value in the present investigation, will forward them to his address, or inform him of their existence, and how they may be made available.

It is also hoped that every person, properly prepared to keep Meteorological registers, will spare no pains to make them as perfect as possible, and not fail to embrace Hygrometrical observations in their tables.

Tables now forming may be of real service to the Committee before the time expires for which it was appointed.

All data received prior to March 15th, 1859, will receive the attention of the Committee; and due credit will be giver in every instance for valuable information.

The subjects of investigation are of such vast interest to mankind, it is hoped the present request will elicit the hearty co-operation of the medical profession, and any other persons who may have, or shall hereafter collect matter of the kind requested.

It would seem that he should not hope in vain, when it is considered that cholera is a disease which knows no political bounds, and frequently appears to regard neither geographical lines or sanitary cordons, and has already destroyed nearly, if not altogether, one hundred millions (100,000,000) of the earth's inhabitants.

Thus far, all with whom the Chairman has corresponded have most promptly proffered any assistance in their power, and at the same time expressed their warmest sympathy and regard for the investigation; but, believing those with whom he is unacquainted may have data of the kind sought, he takes the present method of presenting his wishes, hoping they may be complied with by all who are possessed of such data, and feel an interest in man's emancipation from disease.

Medical Journals, Literary Magazines, and newspapers friendly to the great interests of humanity and medical science, will confer a favor upon the Committee—and it is hoped upon the world—by inserting the above in their columns.

T. WINSLOW GORDON, M.D.

GEORGETOWN, Brown County, O. October, 1856.

—*Correspondence Western Lancet.*

